# Virome Analysis of *Aconitum carmichaelii* Reveals Infection by Eleven Viruses, including Two Potentially New Species

**DOI:** 10.3390/ijms242115558

**Published:** 2023-10-25

**Authors:** Jie Yang, Ping-Xiu Lan, Yun Wang, Jin-Ming Li, Ruhui Li, Steve Wylie, Xiao-Jiao Chen, Gen-Hua Yang, Hong Cai, Fan Li

**Affiliations:** 1 State Key Laboratory for Conservation and Utilization of Bio-Resources in Yunnan, Yunnan Agricultural University, Kunming 650201, China; 2 USDA-ARS, National Germplasm Resources Laboratory, Beltsville, MD 20705, USA; 3 Plant Biotechnology Research Group (Virology), Western Australian State Agricultural Biotechnology Centre, Murdoch University, Murdoch, WA 6150, Australia

**Keywords:** traditional Chinese medicine, virus discovery, virome, high-throughput sequencing, *Potyvirus aconiti*, *Betapartitivirus aconiti*

## Abstract

*Aconitum carmichaelii* is a herbaceous herb indigenous to China that has been cultivated for traditional medicine for centuries. Virus-like symptoms of *A. carmichaelii* plants were observed on leaves in some *A. carmichaelii* plantations in Zhanyi and Wuding Counties, Yunnan Province, southwest China. High-throughput sequencing (HTS) was performed on 28 symptomatic plants, and the results revealed infection with 11 viruses, including 2 novel viruses and 9 previously described viruses: Aconitum amalgavirus 1 (AcoAV-1), aconite virus A (AcVA), cucumber mosaic virus (CMV), currant latent virus (CuLV), apple stem grooving virus (ASGV), chilli veinal mottle virus (ChiVMV), tomato spotted wilt orthotospovirus (TSWV), tobacco vein distorting virus (TVDV), and potato leafroll virus (PLRV). Two novel viruses tentatively named Aconitum potyvirus 1 and Aconitum betapartitivirus 1, were supported by sequence and phylogenetic analysis results of their genomes. We proposed the names *Potyvirus aconiti* and *Betapartitivirus aconiti*. RT-PCR assays of 142 plants revealed the predominance and widespread distribution of CMV, AcVA, and AcoPV-1 in plantations. The detection of isolates of CuLV, ASGV, ChiVMV, TSWV, TVDV, and PLRV infections for the first time in *A. carmichaelii* expands their known host ranges.

## 1. Introduction

*Aconitum* is a large genus comprising approximately 400 species classified within the family *Ranunculaceae*. These species are primarily distributed in Eurasia [1]. They have been of interest since ancient times due to the presence of diterpene alkaloids, which exhibit a wide range of toxicity levels, varying from relatively non-toxic to highly toxic [2]. A notable active compound found in *Aconitum* is aconitine, which is a potent neurotoxin when ingested or absorbed through the skin [3]. Throughout history, *Aconitum* plants have been utilized medicinally and as a source of poisons in various regions around the world, including China, Japan, Korea, and India. *Aconitum carmichaelii* is a species of the genus *Aconitum* and commonly known as Chinese aconite, Carmichael’s Monkshood, Fuzi, and Chuanwu [3]. The drugs made from *A. carmichaelii* tap roots are typically used as painkillers and antirheumatic agents [2]. The lateral roots are commonly used in combination with other herbs to treat conditions such as shock resulting from acute myocardial infarction, low blood pressure, coronary heart disease, and chronic heart failure [4].

*A. carmichaelii* is cultivated for traditional medicine in Asian countries, particularly in China. The commercial production areas are primarily located in Sichuan, Shaanxi, Hubei, Hunan, and Yunnan Provinces [1]. Among these regions, the longest period of *A. carmichaelii* cultivation dates back more than a thousand years in Jiangyou City, Sichuan Province. Since 2015, *A. carmichaelii* cultivation has expanded to approximately 3000 hectares in Shaanxi and Sichuan Provinces, establishing it as a significant local industry [5]. *A. carmichaelii* plants are cultivated through seed and by vegetative propagation from lateral roots. The latter method is preferred for maintaining the genotype and stronger plants [6], which facilitates the accumulation of viruses. Cucumber mosaic virus (CMV) was reported to infect *Aconitum* spp. in 1998 [7], and it remains a predominant virus in *Aconitum* spp. [7,8]. In 2000, Aconitum latent virus (AcLV) was identified in *Aconitum napellus* [7]. With the utilization of high-throughput sequencing (HTS) techniques, aconite virus A (AcVA) [9] and Aconitum amalgavirus 1 (AcoAV-1) [10] were identified.

High-throughput sequencing (HTS) was used to identify the viruses infecting *A. carmichaelii* plants collected from plantations in Zhanyi and Wuding Counties of Yunnan Province in southwest China with the aim of developing diagnostic assays to facilitate the production of a virus-free germplasm of *A. carmichaelii* and enhance viral disease control.

## 2. Results

### 2.1. Viruses Detected in A. carmichaelii Plants

**Figure 1:**
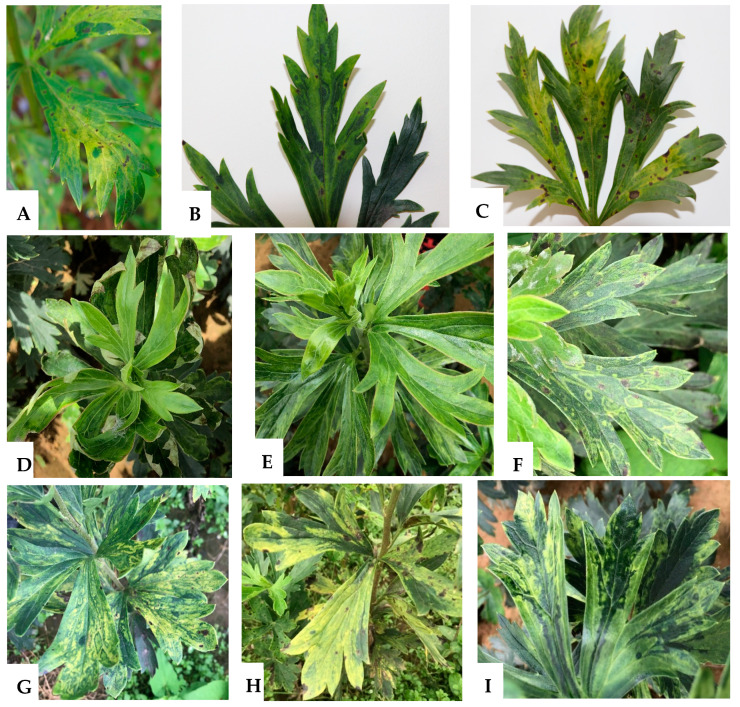
Virus-like symptoms observed on *A. carmichaelii* leaves: (**A**) yellowing, (**B**) ringspot and necrosis, (**C**) necrosis and yellowing, (**D**) leafroll, (**E**) mosaic and mottle, (**F**,**G**) chlorotic ringspot, (**H**) yellowing, and (**I**) chlorosis. Samples (**A**–**C**) were collected from Zhanyi County, and the others (**D**–**I**) were collected from Wuding County.

Virus-like symptoms of ringspot, necrosis, yellowing, mosaic, and mottle were observed on *A. carmichaelii* plants in some open-air plantations in Zhanyi and Wuding Counties, Yunnan Province, southwest China (Figure 1). To identify virus species infecting *A. carmichaelii*, two pooled *A. carmichaelii* leaf tissue samples collected from Zhanyi County in 2017 (comprising 17 individual samples) and 11 of 71 samples collected from Wuding County in 2020 were mixed separately. The two mixed samples were labeled ZY-FZ and WD-FZ, respectively, and subjected to high-throughput sequencing (HTS) after RNA extraction and cDNA synthesis.

There were 13,635,451 and 53,470,900 paired-end reads and 14,423 and 263,871 contigs of >200 nucleotides (nt) in the HTS libraries of ZY-FZ and WD-FZ, respectively. These were assembled de novo from the two libraries. BLASTx and BLASTn analyses revealed the presence of isolates of CMV, AcVA, apple stem grooving virus (ASGV), ChiVMV, TSWV, TVDV, PLRV, and three other virus-like sequences (Table S1). The amino acid sequences of the three virus-like sequences were deduced and shared the greatest amino acid (aa) sequence identities with the genera of some potyviruses (56% of tulip breaking virus), betapartitiviruses (77% of white clover cryptic virus 2 RNA1 and 72% of cannabis cryptic virus RNA2), and cheraviruses (75% of currant latent virus on RNA1 and RNA2). The nearly complete genome sequences of new isolates of CMV, ASGV, ChiVMV, and TSWV detected by HTS data all shared >97% aa sequence identity with available sequences of these viruses, each above the species delineation limit (https://ictv.global/, accessed on 1 December 2020). Two polerovirus-like sequences exhibited >88% aa sequence identity with TVDV and PLRV, respectively, specifically in their RTD regions (Table S1). Subsequent RT-PCR detection confirmed that all the amplicons were identical to CMV, ASGV, ChiVMV, TSWV, TVDV, and PLRV. A carlavirus-like sequence of 5428 nt was closely aligned to an isolate of AcVA (MN944106), sharing 69% nt sequence in their partial *RdRp* genes. Using the AcVA sequence as a template, the HTS data were aligned, and the complete genome sequence of a carlavirus was obtained. The CPs of the carlavirus and the AcVA isolate shared 95% amino acid identities.

### 2.2. Virus Particles in the Symptomatic A. carmichaelii Samples

**Figure 2:**
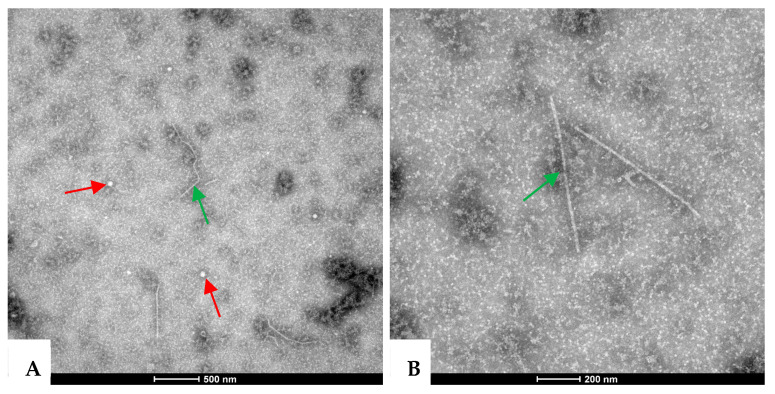
Morphology of virions observed on the diseased *A. carmichaelii* plants under transmission electron microscopy. Spherical virions with a diameter of about 25–30 nm (**A**) and filamentous particles of 600–800 nm (**A**,**B**) in length are indicated by red and green arrows, respectively.

Two types of viral particles—spherical and filamentous in shape—were observed under TEM in the leaf extracts from symptomatic *A. carmichaelii* samples. The spherical particles were 25–30 nm in diameter (Figure 2A), while the filamentous particles were 600–800 nm in length (Figure 2A,B). These results indicate that the presence of viruses with both spherical and filamentous shapes may be associated with diseased *A. carmichaelii* plants.

### 2.3. A Novel Potyvirus Infecting A. carmichaelii Plants

**Figure 3:**
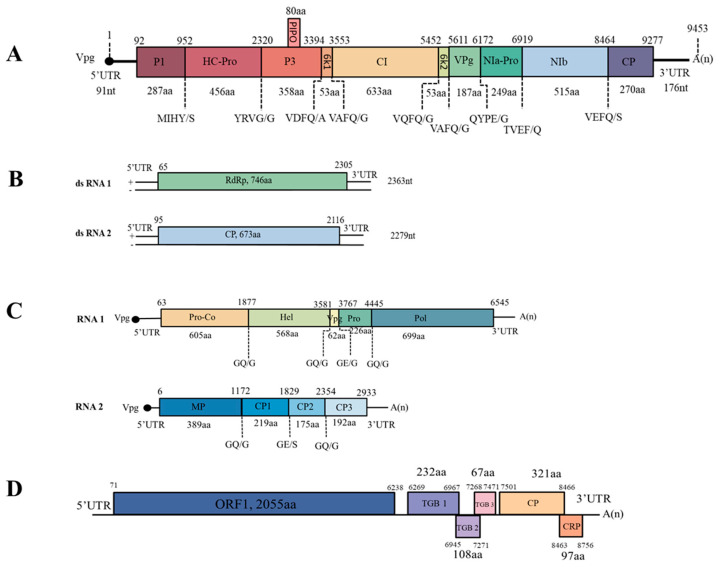
Genome organization of four viruses infecting *A. carmichaelii*. (**A**) Genome organization of Aconitum potyvirus 1; nine highly conserved proteolytic cleavage sites in the polyprotein of AcoPV-1 were predicted using multiple alignment with isolates of PVY, TBV, TuMV, PPV, ParV1, and ParV1. (**B**) Genome organization of Aconitum betapartitivirus 1. (**C**) Genome organization of currant latent virus isolate YWDi. (**D**) Genome organization of aconite virus A isolate YWDi. Different colors are used to distinguish different gene coding products.

A potyvirus-like sequence of 9479 nt identified from the ZY-FZ library was found to be related to known potyviruses. We tentatively propose the novel virus’s name as Aconitum potyvirus 1 (AcoPV-1) isolate YZYi. The complete genomic sequence was obtained through sequence assembly of the amplicons generated from RT-PCR and 5′ RACE reactions. The complete genomic sequence of AcoPV-1 (GenBank Accession MZ389235) is 9453 nt, excluding the 3′ terminal poly (A) tail, in which the 5′ and 3′ untranslated regions (UTRs) are 91 nt and 176 nt, respectively. In common with other potyviruses, AcoPV-1 encodes a large ORF, and the polyprotein is predicted to comprise 3061 aa residues. Nine highly conserved proteolytic cleavage sites in the polyprotein were identified by comparison with the consensus protease recognition motifs of selected potyviruses, which resulted in ten putative mature proteins of P1 (287 aa), HC-Pro (456 aa), P3 (358 aa), 6K1 (53 aa), CI (633 aa), 6K2 (53 aa), VPg (187 aa), NIa-Pro (249 aa), NIb (515 aa), and CP (270 aa) (Figure 3A). The small ORF PIPO within the P3 of potyviruses was also identified by the presence of GA_6_ in AcoPV-1 (nt 2794–2800).

**Figure 4:**
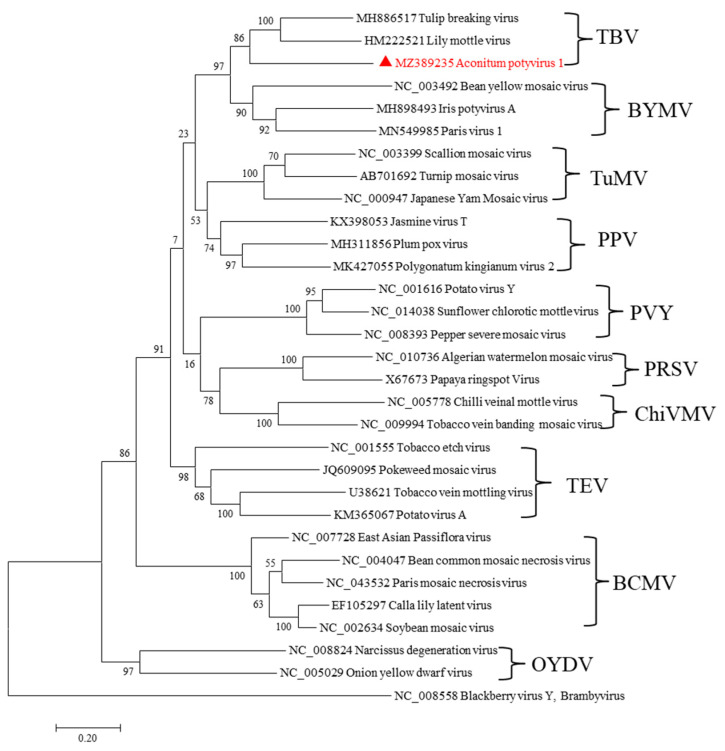
Phylogenetic relationships of Aconitum potyvirus 1 with isolates of other potyviruses based on amino acid sequences of the polyprotein. Each of the subgroups is indicated by an abbreviation of the representative virus and includes some members. The percentage of replicate trees in which the associated taxa clustered together in the bootstrap test (1000 replicates) is shown next to the branches. The scale bar represents a genetic distance of 0.2 substitutions per site. AcoPV-1, as characterized in this study, is indicated by a red triangle. Blackberry virus Y, a potyvirid of the genus *Brambyvirus*, was used as an outgroup.

A pairwise comparison was performed between the complete genomic and polyprotein sequences of AcoPV-1 and those of other potyviruses. AcoPV-1 shares genomic sequence identity below 57% and polyprotein sequence identity below 55% with other potyviruses. All these values fall below the current species delineation criteria for potyvirids, with <76% nt identity with the complete genome and <80% aa identity with the polyprotein [11]. Phylogenetic analysis places AcoPV-1, TBV, and LMoV in the tulip breaking virus (TBV) subgroup (Figure 4).

Most conserved motifs were found in the AcoPV-1 sequence by comparing the deduced polyprotein sequences with those of other known potyviruses [12]. These motifs include ^195^HX_8_DX_33_SGX_22_RG^263^ (proteolytic activity) in P1; ^312^CX_8_CX_18_CX_2_C^343^ (putative zinc finger binding motif), ^602^IGN^604^ (cell-to-cell and long-distance movement), ^466^FRNKX_12_CDNQLD^487^ (symptomatology), ^577^CCCVT^581^ (long distance movement), and ^627^GYCY^630^ (cysteine proteinases) in HC-Pro; four motifs, including ^1355^KVSATPP^1361^, ^1406^LVYV^1409^, ^1457^VATNIIENGVTL^1468^, and ^1501^GERIQRLGRVGR^1512^, related to potential helicase activity in CI; ^2080^HX_34_D_67_GXCGX_14_H^2201^ (proteolytic activity) in NIa-Pro; four motifs, including ^2447^SLKAEL^2452^, ^2480^CVDDFN^2485^, ^2584^GNNSGQPSTVVDNTIMV^2600^, and ^2626^GDD^2629^, related to RNA-dependent polymerase activity in NIb; and ^2975^YMPRYG^2980^, ^2994^AFDF^2997^, and ^3014^QMKAAA^3019^ motifs in CP. Additionally, three motifs associated with aphid transmission—^337^RITC^340^ and ^595^PTK^597^ in HC-Pro and ^2797^DAG^2799^ in CP—were found in the AcoPV-1 polyprotein sequence.

The abovementioned analysis results support the classification of Aconitum potyvirus 1 isolate YZYi as a novel member of the *Potyvirus* genus. We propose the name *Potyvirus aconiti* for this new species.

### 2.4. A Novel Betapartitivirus in A. carmichaelii Plants

**Figure 5:**
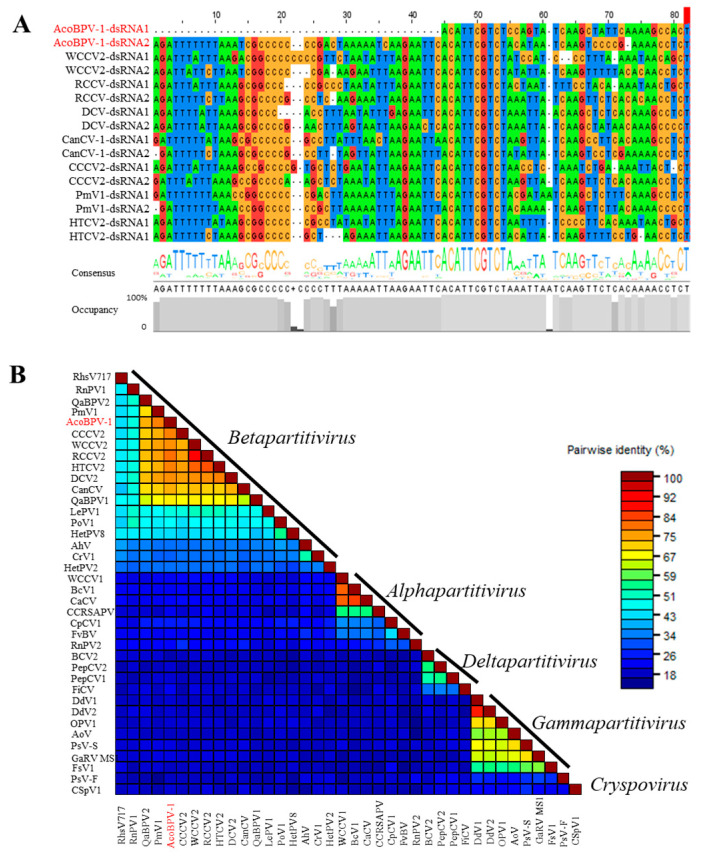
Genome sequence analysis of Aconitum betapartitivirus 1. (**A**) The conserved nucleotide sequences are present at the 5’ termini of dsRNA1 and dsRNA2 in AcoBPV-1and the other seven betapatitiviruses. Multiple sequence alignment was conducted using Jalview software 2.11. The same color indicates the identical nucleotide. (**B**) Pairwise identity plot of CPs in the *Partitiviridae* family aligned using the MAFFT program and displayed using Sequence Demarcation Tool (SDT) v1.2 software.

Two contigs, one of 2434 nt and the other of 2244 nt, were assembled from the WD-FZ library. The sequences showed the highest nt sequence identities with betapartitiviruses. This putative virus was named Aconitum betapartitivirus 1 (AcoBPV-1) isolate YWDi. Subsequently, the two RNA segments of AcoBPV-1 were obtained by RT-PCR and 5′ RACE assays using the primers listed in Table S8, followed by sequence assembly. The near-complete RNA1 sequence was 2363 nt (GenBank Accession OR096294), excluding the 3′ terminal poly (A) tail. Attempts to obtain the terminal bases of the 5′ UTR of RNA1 using 5′ RACE were unsuccessful. The complete sequence of RNA2 was 2279 nt (GenBank Accession OR096295), excluding its poly (A) tail. Each of the two RNA segments is predicted to encode one ORF (Figure 3B). RNA1 has a putative ORF (ORF1) starting at nt 65 (AUG) and ending at nt 2303–2305 (UGA) on its plus strand, potentially encoding an 86.6 kDa protein of 746 aa. According to the NCBI conserved domain database, there is a domain matching RNA-dependent RNA polymerase (RdRp) in aa positions 342–602 (E-value: 3.92 × 10^−6^); therefore, ORF1 probably encodes for an RdRp. RNA2 has a putative ORF (ORF2) starting at nt 95 (AUG) and terminating at nt 2114–2116 (UAG), encoding a putative 673 aa protein with a molecular weight of 74.89 kDa. A BLASTp search revealed that the two predicted proteins are closely related to RdRps and CPs of betapartitiviruses, which are members of the *Partitiviridae* family. The RdRp aa sequence was 80.2% identical to that of red clover cryptic virus 2 (RCCV2, NC_021096), and dill cryptic virus 2 (DCV2, NC_021148) was found to be the most closely related virus to CP, with 71.2% aa sequence identity. All these values fall below the current species delineation criteria for the *Betapartitivirus* genus, which require ≤90% aa sequence identity in the RdRP and ≤80% aa sequence identity in the CP [13]. Many dsRNA viruses have conserved 5′-terminal and 3′ terminal sequences, which are generally thought to be involved in RdRp recognition or RNA packaging [14]. The 5′ UTRs of AcoBPV-1 RNA1 and RNA2 are 64 nt (incomplete) and 95 nt, with 3′ UTRs of 58 nt and 163 nt, respectively. An “AGATTTTTT” sequence was found at the 5′ terminus in the dsRNA2 of AcoBPV-1, which is conserved in betapartitiviruses (Figure 5A). The aligned sequences show that the 5’ terminus of AcoBPV-1 dsRNA1 lacks a 44 nt fragment, and it remains to be seen whether the missing region contains the AGATTTTTT sequence.

**Figure 6:**
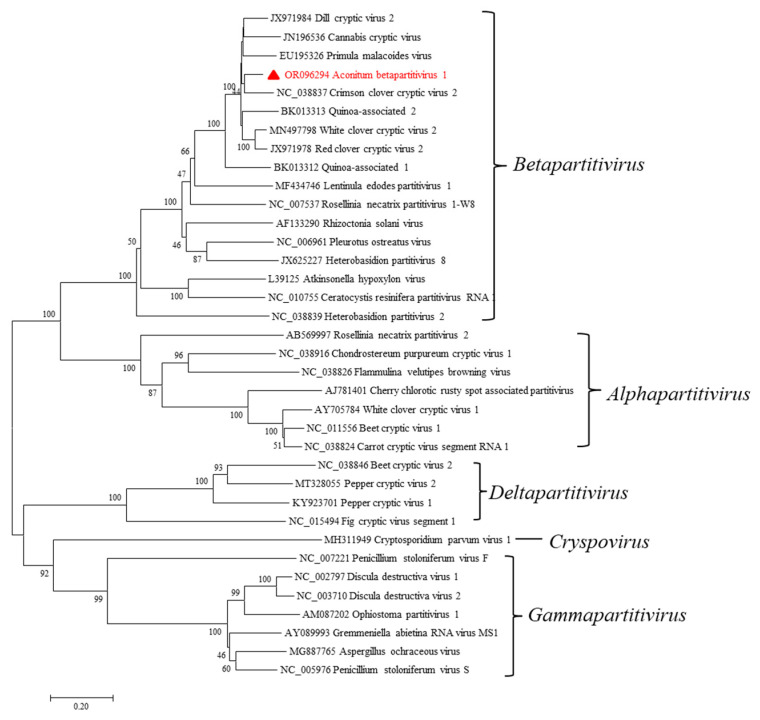
Phylogenetic relationships of Aconitum betapartitivirus 1 and other members of the *Partitiviridae* family based on RNA-dependent RNA polymerase (RdRp) amino acid sequences determined using MEGA7 according to the maximum-likelihood method with 1000 bootstrap replicates. The scale bar represents a genetic distance of 0.2. AcoBPV-1, as characterized in this study, is indicated by a red triangle.

AcoBPV-1 classification status was further determined using its genome sequence assembled from RT-PCR and 5’ RACE amplicons. The CP aa sequences of all partitiviruses were aligned using the MAFFT program, and pairwise identities were visualized with SDT v1.2 software. The color-coded matrix plots show that pairwise identities can be categorized into five groups, which correspond to the five genera in *Partitiviridae*. AcoBPV-1 clusters within the betapartitiviruses (Figure 5B). Based on RdRp amino acid sequence alignments, the newly identified AcoBPV-1 clusters closest to CCCV2 within a monophyletic clade consisting entirely of betapartitiviruses (Figure 6).

Based on the phylogenetic results described above and the other findings, AcoBPV-1 exhibited many characteristics of betapartitiviruses. Consequently, we propose that Aconitum betapartitivirus 1 (AcoBPV-1) isolate YWDi can be considered a new candidate member in *Betapartitivirus* genus, for which we propose the species name *Betapartitivirus aconiti*.

### 2.5. Diversity Analysis of Currant Latent Virus

A putative viral genome with two RNA segments, one of 6712 nt and the other of 2270 nt, was assembled from the WD-FZ library. This virus showed the closest genomic identity and shared features with previously described cheraviruses, particularly with CuLV. RT-PCR and 5′ RACE reactions were conducted in an attempt to obtain the complete RNA1 and RNA2 sequences of the putative cheravirus (isolate YWDi). Unfortunately, the full 5′ UTR sequence of RNA2 was not obtained after several 5′ RACE attempts. The proposed RNA1 (GenBank Accession OR096292) and RNA2 (GenBank Accession OR096293) of the cheravirus YWDi isolate were 6683 nt and 3070 nt long, respectively, excluding poly (A) tails. The complete or near-complete 5′ UTR of RNA1 was 62 nt, and 5 nt was obtained for the 5′ UTR of RNA2 (likely incomplete). The 3′ UTR sequences of RNA1 and RNA2 were 141 nt and 140 nt long, respectively. Like other members of the genus *Cheravirus*, the 3′ terminal sequences of the two RNAs also have a high sequence identity of 87.3% [15].

**Figure 7:**
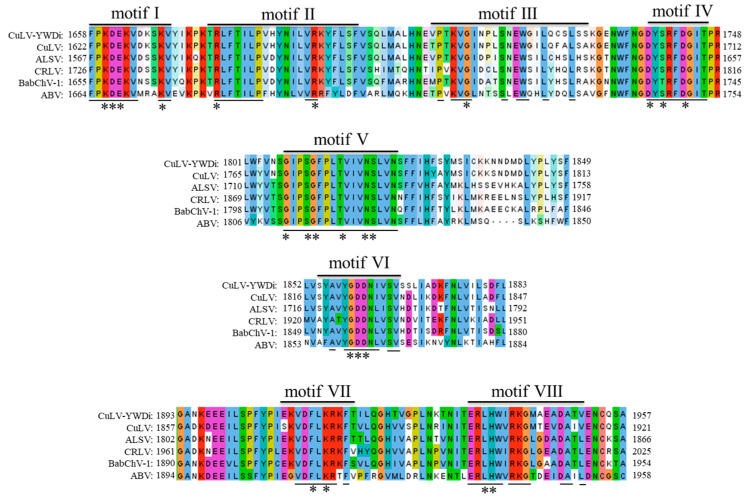
Amino acid sequence alignment between the conserved motifs of RNA-dependent RNA polymerases (RdRps) of currant latent virus (CuLV) isolate YWDi and other cheraviruses. Multiple sequence alignment was conducted using Jalview software 2.11. *, **, *** indicate the conserved amino acids in different motifs. The same color indicates the identical amino acid.

RNA1 and RNA2 were predicted to encode a single polyprotein of 2160 aa (243.28 kDa) and 975 aa (108.32 kDa), respectively (Figure 3C). By analogy to the structures of the polyproteins from apple latent spherical virus (ALSV) [16], cherry rasp leaf virus (CRLV) [17], arracacha virus B (AVB) [18], CuLV [19], and stocky prune virus (StPV) [20], the polyprotein encoded from RNA1 appears to be cleaved into five different mature proteins: a protease cofactor (Pro-Co, aa 1–605), a helicase (Hel, aa 606–1173), a VPg (aa 1174–1235), a serine protease (Pro, aa 1236–1461), and an RdRp (aa 1462–2160), with specific cleavage sites of GQ/G, GQ/G, GE/G, and GQ/G, respectively. The Pro-Co protein contains an ^480^FX_22_W_34_L^538^ (F-W-L) motif, unlike the FX_26_WX_34_L motif identified in ALSV and the FX_30_WX_15_LX_20_L motif found in AVB [16,18]. The Hel protein has GKS (aa 790–792), DE (aa 836–837), and N (aa 859) domains, which are prevalent in +ssRNA viruses [21]. The expected H (aa 1278), D (aa 1316), and C (aa 1411) motifs were also found in the Pro protein. Additionally, the RdRp protein has all eight typical motifs of members of the *Cheravirus* genus (Figure 7) [22]. Similar to other cheraviruses, the RNA2 polyprotein consists of an MP (movement protein, aa 1–389) and three CPs (CP1, aa 390–608; CP2, aa 609–783; and CP3, aa 784–975—with cleavage sites of GQ/G, GE/S, and GQ/G, respectively). A conserved-domain LPL (aa 287–289) was found in the predicted MP.

**Figure 8:**
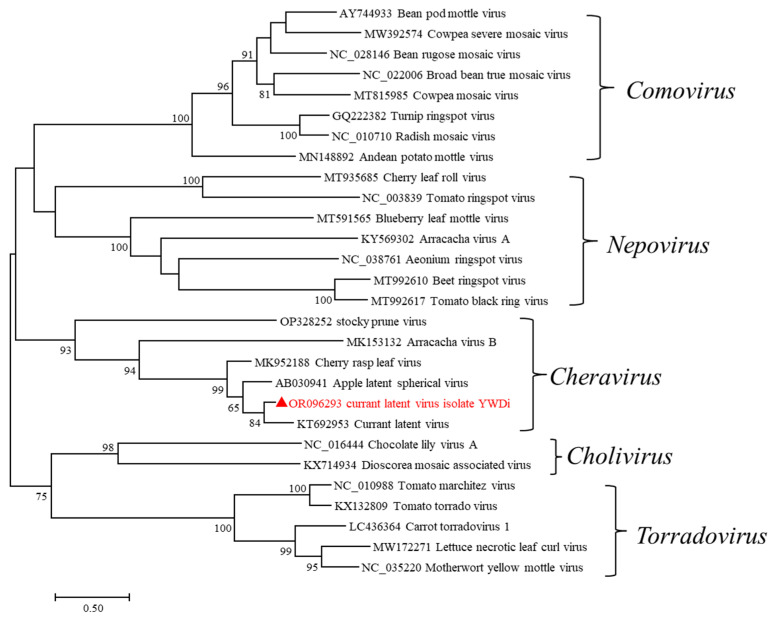
Phylogenetic relationships of currant latent virus isolate YWDi (indicated by a red triangle) with other members of the *Secoviridae* family based on their amino acid sequences of RNA2-encoded polyprotein, which was performed using MEGA7 according to the maximum-likelihood method with 1000 bootstrap replicates. The scale bar represents a genetic distance of 0.5 substitutions per site.

Further sequence analysis was carried out on the genome sequence of cheravirus isolate YWDi, which was assembled from RT-PCR and 5’ RACE amplicons. Pairwise comparisons were conducted between the deduced aa sequences of cheravirus isolate YWDi and those of cheraviruses ALSV, CRLV, AVB, CuLV, and StPV (Table S2). The RNA1 and RNA2 polyproteins of cheravirus isolate YWDi share the highest aa sequence identity with currant latent virus isolates, sharing 79% aa identity in the conserved Pro-Pol region and 77% aa sequence identity in the combined CP. These values are very close to the accepted species delineation criteria of <80% aa identity for Pro-Pol and <75% aa identity for CP for members of the *Secoviridae* family [23]. Phylogenetic analysis based on the RNA2 amino acid sequences of cheraviruses and selected members of the *Secoviridae* was performed using MEGA 7 software. The phylogenetic analysis grouped cheravirus isolate YWDi, along with CuLV, ALSV, CRLV, AVB, and StPV, into a cluster of cheraviruses (Figure 8). These results suggest that cheravirus isolate YWDi is a divergent isolate of currant latent virus. Therefore, we named it currant latent virus isolate YWDi.

### 2.6. Diversity Analysis of Aconite Virus A

A carlavirus-like sequence of 5428 nt was assembled from the WD-FZ library. It was closely aligned to an isolate of AcVA (MN944106). Using the AcVA sequence as a template, RT-PCR and 5′ RACE reactions were conducted to obtain the complete genome sequence of the carlavirus. The putative carlavirus (isolate YWDi) was 8833 nt in length, excluding the poly (A) tail at the 3′ end. Genome sequence analysis revealed that the new sequence contained six putative ORFs and possessed a genomic structure and sequence typical of carlaviruses (Figure 3D). The complete sequence of carlavirus isolate YWDi shared the greatest nt sequence identity (73%) with aconite virus A (AcVA) isolate HB (GenBank Accession MN944106). The ORF1 (nt 71–6238) of isolate YWDi follows a short 5′ UTR of 70 nt and is predicted to encode a viral replicase of 2055 aa residues, and a conserved motif (^1896^SGX_3_TX_3_NTX_22_GDD^1931^) was identified near the C terminus of the RdRp domain. Three overlapping ORFs—ORF2 (nt 6269–6967), ORF3 (nt 6945–7271), and ORF4 (nt 7268–7471)—were predicted to encode triple gene block (TGB) proteins TGBp1 (232 aa), TGBp2 (108 aa), and TGBp3 (67 aa), respectively. ORF5 (nt 7501–8466) encodes a CP of 321 aa residues. A zinc-finger-like motif (^56^CX_2_CX_12_CX_4_C^77^) and a nuclear localization signal (NLS) (^46^RKRR^49^) were found in the core region of the cysteine-rich protein (CRP) encoded by ORF6 (nt 8463–8756) with 97 aa residues.

Comparing the ORFs of carlavirus isolate YWDi and AcVA isolate HB, the lengths of ORF2-ORF6 were identical, except that the ORF1 in isolate YWDi had two amino acid residues fewer than AcVA isolate HB. Pairwise comparisons between isolates YWDi and HB revealed that their complete genomes share 71.8% nucleotide sequence identity. The five ORFs (ORF1, TGBp1, TGBp2, TGBp3, and CRP) of the two isolates share 71.3%, 69.1%, 65.7%, 67.6%, and 81% nt sequence identities, respectively, while at the aa level, they share 79.6%, 72%, 75.9%, 55.2%, and 80.4% sequence identities, respectively. In particular, the two isolates were found to share 79.1% nt sequence identity and 95.3% aa sequence identity in their CPs. According to the species delineation threshold for the *Carlavirus* genus of <72% nt or <80% aa sequence identity in the CP or polymerase [24], we propose that isolate YWDi identified in this study can be classified as a divergent isolate of AcVA.

**Figure 9:**
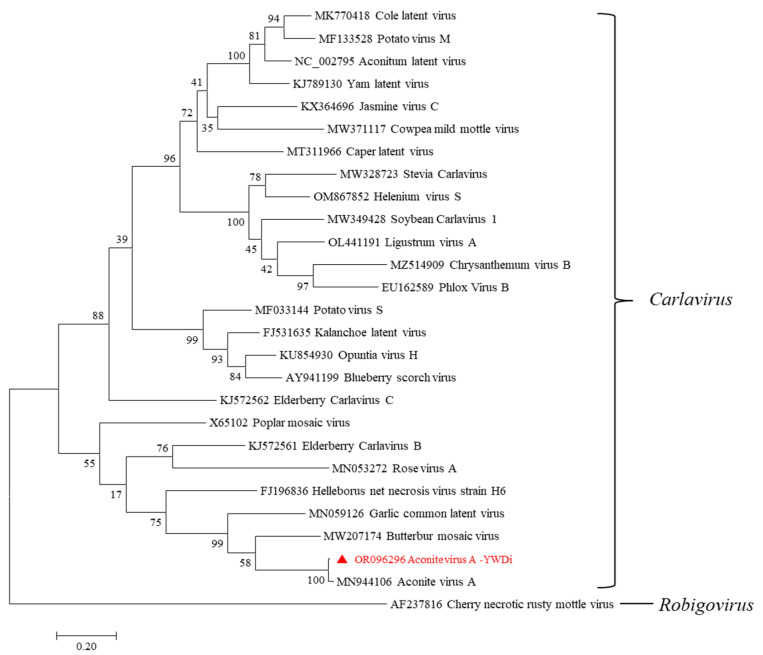
Maximum-likelihood tree based on CP amino acid sequences of AcVA-YWDi and other carlaviruses. Phylogenetic analysis was applied using 1000 bootstrap replicates. The scale bar represents a genetic distance of 0.2. AcVA-YWDi, as characterized in this study, is indicated by a solid red triangle. Cherry necrotic rusty mottle virus, a member of the *Robigovirus* genus, was used as an outgroup.

Phylogenetic analysis was performed using the maximum-likelihood method with 1000 bootstrap replicates in MEGA7. A phylogram was generated based on the aa sequences of CPs of related carlaviruses. The phylogenetic relationship shows that AcVA-YWDi and AcVA-HB cluster together with other carlaviruses (Figure 9). Sites of possible recombination events within the genome sequences of AcVA-YWDi, AcVA-HB, and 25 other carlaviruses were tested using the RDP4 version 4.9 package with default settings and RDP, Chimaera, BootScan, 3Seq, GENECONV, MaxChi, and SiScan programs. A recombination event was detected by SiScan with a P-value of 2.535 × 10^−39^ at nt 372–5453 in the genome of AcVA-HB, with rose virus A (RVA, MN053272) as a potential major parent and cowpea mild mottle virus (CPMMV, MW371117) as a potential minor parent (Figure S2).

Based on sequence comparisons and phylogenetic and recombination analysis, we confirm that the carlavirus identified in this study is a distinct isolate of aconite virus A, which we named aconite virus A isolate YWDi (GenBank Accession OR096296).

### 2.7. Incidence of Eleven Viruses in Aconitum carmichaelii Plants

Virus-specific primers were designed to determine the incidence of each of 11 viruses in *A. carmichaelii* from Zhanyi and Wuding Counties (Table S3). All 142 leaf samples were individually used for total RNA extraction and subsequent testing for each of the target viruses. Based on the sequencing results of the target amplicons, except for AcLV, isolates of eleven viruses—AcoPV-1, AcoBPV-1, AcoAV-1, AcVA, CuLV, CMV, ASGV, PLRV, ChiVMV, TVDV, and TSWV—were identified in *A. carmichaelii* samples (Figure S1, Table 1).

Of the 142 *A. carmichaelii* samples, 132 had single or mixed viral infections (92.9%) (Table 2 and Table S4). Three of the eleven identified viruses—CMV, AcoPV-1, and AcoAV-1— were found in both counties, while five others—AcVA, AcoBPV-1, CuLV, ASGV, and ChiVMV—were identified only in Wuding County, and three viruses—TVDV, TSWV, and PLRV—were found only in Zhanyi County. CMV, AcVA, and AcoPV-1 were the most predominant viruses (Table 2).

There were one to seven single or mixed virus infections on *A. carmichaelii* plants (Tables S4 and S5). Among them, four-virus mixed infection was most common (28.2%), followed by three- (23.9%) and five- (15.5%) virus coinfections. Of 49 different single or mixed infection types in the diseased *A. carmichaelii* plants, mixed infections occurred mainly with the involvement of CMV, AcVA, and AcoPV-1 (Table S5). The coinfection AcoPV-1 + ACVA + CMV, AcoPV-1 + CMV, AcoPV-1 + AcoBPV-1 + ACVA + CMV, and AcoPV-1 + AcoAV-1 + ACVA + CMV coinfection types had the highest detection rates of 8.5%, 5.6%, 5.6%, and 5.6%, respectively.

### 2.8. Seed Transmission of CMV, AcoPV-1, AcVA, and AcoAV-1

Among the 15 *A. carmichaelii* plant seeds collected from the field and planted in an insect-proof greenhouse, 2 exhibited mottle symptoms after two months of germination (Figure S3A,B), while the remaining 13 seedlings remained asymptomatic (Figure S3C). All these plants were subjected to RT-PCR using specific primers of the 11 viruses (Table S3), and CMV, AcoPV-1, AcVA, and AcoAV-1 were detected in the tested *A. carmichaelii* plants (Figure S4). Therefore, CMV, AcoPV-1, AcVA, and AcoAV-1 may be transmitted by the infested *A. carmichaelii* seed. However, it is not possible to determine whether the remaining seven viruses can be transmitted through the seeds of aconite, as it is unclear whether the 15 seeds used for testing carry CuLV, AcoBPV-1, ASGV, TVDV, TSWV, TSWV, and PLRV.

## 3. Discussion

Here, we identified eleven viruses infecting *A. carmichaelii* plants in two growing regions in Yunnan Province, China. Sequencing analysis revealed isolates of viruses belonging to nine viral genera, namely AcoPV-1 and ChiVMV in the *Potyvirus* genus, AcoBPV-1 in *Betapartitivirus* genus, CuLV in *Cheravirus* genus, AcoAV-1 in *Amalgavirus* genus, AcVA in *Carlavirus* genus, CMV in *Cucumovirus* genus, ASGV in *Capillovirus* genus, PLRV and TVDV in *Polerovirus* genus, and TSWV in the *Orthotospovirus* genus. Among the eleven identified viruses, two viruses—AcoPV-1 and AcoBPV-1—were novel, and six viruses, namely CuLV, ASGV, PLRV, ChiVMV, TVDV, and TSWV, were identified in *A. carmichaelii* for the first time. Only AcoAV-1, AcVA, and CMV were previously reported in the plant [8,9,10]. These data indicate the rich diversity of viruses infecting *A. carmichaelii* plants.

During this investigation, we found that viral diseases occurred in all *A. carmichaelii* fields, and there was no significant difference in symptoms. The majority of diseased plants were infected by at least one virus. The viral incidence between the two counties showed significant differences. Six viruses—AcoPV-1, AcoAV-1, CMV, PLRV, TVDV, and TSWV—were detected in Zhanyi, while eight viruses—AcoPV-1, AcoBPV-1, AcoAV-1, CMV, CuLV, AcVA, ASGV, and ChiVMV—were detected in Wuding. Only AcoPV-1, AcoAV-1, and CMV were present in both counties, with AcoPV-1 and CMV being the most prevalent viruses in both areas. Furthermore, PLRV, TVDV, and TSWV were only found in the samples collected from Zhanyi, and ChiVMV was exclusively detected in the samples collected from Wuding in 2020 (Table 2). The apparent differences in viruses between regions and years may not be real and are probably related to the relatively low numbers of samples collected.

AcVA-YWDi exhibited 71.8% nt genome sequence identity with isolate AcVA-HB. The *RdRp* and *cp* genes displayed nt sequence identities of 71.3% and 79.1% and aa sequence identities of 79.6% and 95.3%, respectively. The two AcVA isolates demonstrated significant genetic divergence, although they did not meet the criteria for defining a new species. It remains unclear whether these molecular variations result in any discernible biological differences between the two AcVA isolates. This study offers novel insights into the wide genetic diversity of this virus, presenting information about both conserved and variable regions of its genome. Such data hold significant importance in the development of PCR-based assays for AcVA, particularly in primer design, as well as for future studies on the molecular biology of the virus.

CuLV-YWDi exhibits the characteristic genomic structure of *Cheravirus* and shares 79% aa identity in the conserved Pro-Pol region and 77% aa sequence identity in the combined CP region with isolates of CuLV (GenBank Accessions KT692952 and KT692953). These values closely align with the established species delineation criteria for members of the *Secoviridae* family [23]. Without conducting any biological property tests, the classification of CuLV-YWDi as a novel cheravirus lacks sufficient evidence. We suggest that the YWDi isolate identified as a cheravirus could potentially represent a diverse isolate of CuLV.

An increase in disease incidence and a decrease in yield have coincided with the expansion of the cultivation and continuous cropping of *A. carmichaelii* in recent years. Meanwhile, viral disease has progressively emerged as a limiting factor, posing a threat to the advancement of aconite cultivation [7,8,9,10]. This study reveals a significantly high incidence of viruses in *A. carmichaeli* plants, with a detection rate of 93% (Table S4), indicating a severe epidemic of viral diseases in *A. carmichaeli* plantations in Yunnan Province, China.

Members of *Potyvirus* [25], *Partitivirus* [13], *Cheravirus* [26,27], *Carlavirus* [28], and *Amalgavirus* [10,29] have been reported as seed-borne. In this study, AcoPV-1, AcVA, CMV, and AcoAV-1 have also been found in seedlings germinated from *A. carmichaelii* plant seeds. Viruses identified in this study, such as CMV, AcoPV-1 ASGV, AcoBPV-1, CuLV, AcoAV-1, and AcVA, are likely transmitted through seeds. Like most Chinese medicinal plants, *A. carmichaelii* plants are cultivated from seeds and vegetatively from root tubers [1,6], and both propagation methods could potentially facilitate the transmission of viruses.

Vectors play vital roles in viral long-distance transmission and prevalence [30]. This study identified aphid-borne viruses CMV [31], ChiVMV [32], TVDV [33], and PLRV [34], as well as thrips-borne TSWV [35], infecting *A. carmichaelii* plants. Potyviruses [36], certain cheraviruses [19], and the majority of carlaviruses [37] are transmitted by aphids. The identification of aphid-transmission-related motifs in novel potyvirus AcoPV-1 further suggests its potential transmission by aphids. Further research should assess the prevalence of insect vectors and the incidence of the 11 viruses in other crops and weeds in *A. carmichaelii* growing areas.

Regarding the viruses detected in *A. carmichaeli*, it is well-documented that CMV [38], ASGV [39,40], ChiVMV [41], PLRV [34], TVDV [33,42], and TSWV [38] have the ability to infect other economically important crops. The findings reported in this study suggest that the host range of these viruses is expanding. However, their impact on *A. carmichaelii* remains unknown. Furthermore, there is limited understanding of the host range and transmission ways for AcoPV-1, AcoAV-1, AcoBPV-1, CuLV, and AcVA, which raises concerns that other plant species, apart from *A. carmichaeli*, may be at risk. In future research, it is crucial to conduct comprehensive experiments using multiple methods to uncover the biological and molecular characteristics of AcoPV-1, AcoAV-1, AcoBPV-1, CuLV, and AcVA, as well as to investigate the impact of these 11 viruses on *A. carmichaeli* plants. Most importantly, priority should be given to strengthening virus monitoring, promoting the cultivation of healthy seedlings, and interrupting transmission ways as fundamental strategies for the management of viral disease in *A. carmichaeli* plants.

This study presents the first reports of *A. carmichaeli* infection with CuLV, ASGV, ChiVMV, PLRV, TVDV, and TSWV. It also introduces two previously unreported viruses infecting *A. carmichaeli*: AcoPV-1 and AcoBPV-1, for which we propose two new species. The International Committee on Taxonomy of Viruses (ICTV) recently adopted binomial taxonomic names (*Genus species*) for viruses to align with the classification of cellular life [43]. Consequently, we propose the formal taxonomic names *Potyvirus aconiti* and *Betapartitivirus aconiti* to classify viruses Aconitum potyvirus 1 and Aconitum betapartitivirus 1, respectively.

## 4. Materials and Methods

### 4.1. Plant Material

In October 2017, 17 leaf samples displaying virus-like symptoms of ringspot, necrosis, and yellowing were collected from *A. carmichaelii* plants in Zhanyi County, Yunnan Province, China (Figure 1A–C). During July 2020 and August 2021, a further investigation of viral disease in *A. carmichaelii* was conducted in Wuding County, Yunnan Province. Leaves with symptoms of foliar mosaic, mottle, ringspot, yellowing, leafroll, and chlorosis were observed on plants in Wuding County (Figure 1D–I), and 71 and 54 symptomatic samples were collected from the *A. carmichaelii* plants grown in plantations in 2020 and 2021, respectively. The 142 samples collected in this study were stored at −80 °C until use.

### 4.2. Virus Particle Observation

The negative staining method described by Lan [41] was used for viral diseased *A. carmichaelii* samples, with slight optimization. Leaf tissues from a pooled sample (WD-FZ, comprising 11 out of the 71 samples collected in Wuding County in 2020) were ground into fine powder using liquid nitrogen. Approximately 0.1 g of the powder was transferred to a 1.5 mL microcentrifuge tube containing 200 μL of 200 mM sodium phosphate (NaPO_4_) buffer (pH 6.8). After centrifugation at 12,000 rpm for 10 min at 4 °C, a small drop of the supernatant was placed onto a piece of sealing film. A carbon-coated grid was then inverted and gently pressed onto the drop for 2–3 min. Excess liquid on the grid was removed using filter paper, and the grid was subsequently stained with a drop of 2% phosphotungstic acid (pH 6.8) for 1 min before observation under a transmission electron microscope (TEM) (FEI TECNAI SPIRIT G2 (FEI, Hillsboro, OR, USA)). Images containing virus particles were digitally recorded, and measurements were taken for some of them.

### 4.3. RNA Extraction, HTS, and Data Processing

A pooled sample of the 17 *A. carmichaelii* leaf tissues collected in Zhanyi County labelled as ZY-FZ was subjected to high-throughput sequencing (HTS) after RNA Extraction and cDNA synthesis. The ZY-FZ library was synthesized by BioMarker Biotech Co., Ltd., (Beijing, China) in 2017. Another pooled sample containing 11 of the 71 samples collected in Wuding County in 2020 was named WD-FZ and also prepared for HTS. The WD-FZ library was synthesized by Origingene Bio-Pharm Technology Co. (Shanghai, China) in 2020.

Total RNA was isolated from leaf tissues of the two pooled samples (ZY-FZ and WD-FZ) using Trizol (Invitrogen, Carlsbad, CA, USA) reagent following the manufacturer’s instructions. The purity, concentration, and integrity of RNA were determined by NanoDrop 2000 (NanoDrop, Wilmington, DE, USA) and Agilent 2100 (Agilent Technologies, Santa Clara, CA, USA) until OD260/280 was equal to 1.6–1.8. Ribosomal RNAs were depleted using a RiboZero Magnetic Kit (Epicentre, Lucigen, Middleton, WI, USA). The RNA-seq libraries were constructed using a NEBNext^®^ UltraTM RNA Library Prep Kit (Illumina, San Diego, CA, USA) with random primer (pd(N)9). Enrichment and cleaving of mRNA, synthesis of first-strand cDNA, synthesis and terminal repair of second-strand cDNA, the addition of an A base to the blunt ends of each strand, ligation of cDNA to the indexed adapters, and purification and amplification of the ligation fragments were performed according to the manufacturer’s instructions to prepare the RNA-seq library. The average inserts size was 350 bp (±50 bp), and sequencing was performed by paired-end sequencing on an Illumina HiSeq X-ten platform (LC Bio, Hangzhou, China) following the manufacturer-provided standard protocol. After trimming primer sequences, paired-end reads were obtained and used for de novo assembly. The resulting contigs were subjected to BLASTx and BLASTn searches against viral sequences of local datasets retrieved from the National Center for Biotechnology Information (NCBI) databanks. These processes allowed for the identification of contigs with sequences resembling viral genomes.

### 4.4. Reconstruction of Virus Genomes

Based on the HTS data, seven viruses, namely CMV, AcoAV-1, ASGV, ChiVMV, PLRV, TVDV, and TSWV, exhibited a high sequence identity with the known viruses in GenBank. However, only specific regions of their genomes were amplified for identification following the methods described in Section 4.5.

To verify the presence of AcoPV-1, AcoBPV-1, and the distinct isolates of AcVA and CuLV in *A. carmichaelii* samples, total RNA was prepared from each of the 28 HTS samples (17 from Zhanyi County and 11 from Wuding County) using an EasyPure Plant RNA Kit (TransGen Biotech, Beijing, China). Samples positive for one or more viruses were selected, and four samples were used to amplify the complete genome sequences of AcVA, CuLV, AcoPV-1, and AcoBPV-1. A set of specific primers based on the viral contigs and the known virus sequences was designed to amplify overlapping fragments for each of the four viruses (Tables S6–S9). Evaluation of the primers was conducted on http://biotools.nubic.northwestern.edu/OligoCalc.html (accessed on 8 August 2021). All neighboring primers had an overlapping region of 115–151 nt at the ends of the contiguous amplicons. RT-PCR was performed using a PrimeScript™ One-Step RT-PCR Kit Ver. 2 (TaKaRa Biotechnology Co. Ltd., Dalian, China). The 5′-end sequence was amplified using a SMARTer RACE 5′/3′ kit (TaKaRa Biotechnology) according to the manufacturer’s instructions, whereas their 3′-end sequences were obtained using the method described by Lan et al. [41]. Each of the amplicons obtained using the primers (Tables S6–S9) was cloned into the pMD19-T vector (TaKaRa Biotechnology). At least three clones of each amplicon were sequenced in each direction (Sangon, Shanghai, China).

### 4.5. Virus Incidence

To determine the infection rate of each of the 11 viruses infecting *A. carmichaelii*, all 142 leaf samples from both Counties were used to extract total RNA using either and EasyPure Plant RNA Kit (TransGen Biotech, Beijing, China) or a CTAB-based method [44] and tested individually for each of the target viruses. cDNA synthesis was conducted using M-MLV reverse transcriptase according to the manufacturer’s instructions (TaKaRa Biotechnology). PCR was carried out in GreenTaq Mix (Vazyme Biotech Co., Ltd., Nanjing, China) using virus-specific primers (Table S3) designed according to the genomic sequences obtained from this and previous studies. Positive controls consisted of RNAs extracted from the *A. carmichaelii* plants infected with the target viruses, while the negative control was ddH_2_O. The amplicons were detected using 1% agarose gels in TBE buffer. Bands with the expected size were then excised, purified from the gel, and cloned into the pMD19-T vector (TaKaRa Biotechnology). For each amplicon, sequences were obtained from overnight cultures of at least three clones (Sangon, Shanghai, China).

### 4.6. Genomic and Phylogenetic Analyses

The (full-length) genomes from RT-PCR and Sanger sequencing of AcVA, CuLV, AcoPV-1, and AcoBPV-1 were assembled and analyzed using SeqMan in the Lasergene v7.1 package (DNASTAR Inc., Madison, WI, USA). Phylogenetic analysis was performed in MEGA7 using the maximum-likelihood method with 1000 bootstrap replicates. Viral genomic organizations were analyzed using the ORF finder program (https://www.ncbi.nlm.nih.gov/orffinder, accessed on 12 October 2022). The conserved domains of ORF analysis were using the Conserved Domain Search Service on the NCBI website (www.ncbi.nlm.nih.gov/Structure/cdd/wrpsb.cgi, accessed on 8 December 2022). Pairwise comparisons of nucleotide and amino acid sequences were performed using the Pairwise Sequence Alignment of EMBOSS Needle at https://www.ebi.ac.uk/Tools/psa/emboss_needle/ (accessed on 12 January 2023). Multiple sequence alignment was conducted using Clustal Omega at https://www.ebi.ac.uk/Tools/msa/clustalo/ (accessed on 18 October 2023) and Jalview software 2.11 [45]. Nine highly-conserved proteolytic cleavage sites in the polyprotein of AcoPV-1 were predicted using a multiple alignment with isolates of potato virus Y (PVY, NC_001616), tulip breaking virus (TBV, MH886517), turnip mosaic virus (TuMV, AB701692), plum pox virus (PPV, MH311856), Paris virus 1 (ParV1, MN549985), and lily mottle virus (LMoV, HM222521). Putative dipeptides of cleavage sites in the two polyproteins of CuLV-YWDi were deduced based on alignment with genomes of known cheraviruses. SDT v1.2 software was used to display the pairwise identity of CPs between all of the partitiviruses aligned by the MAFFT program [46]. Recombination analyses of AcVA and other carlavirus genomes were performed in RDP4 using the RDP, Chimaera, BootScan, 3Seq, GENECONV, MaxChi, and SiScan detection methods [47].

### 4.7. Seed Transmission Test of Viruses Identified in A. carmichaelii

From February to April 2022, fifteen A. carmichaelii seeds were collected from the field and planted in an insect-proof greenhouse. After germination, infections with each of 11 viruses were determined in all of these plants using RT-PCR with specific primers (Table S3). The expected amplicons were sequenced following the methods described in Section 4.5.

## 5. Conclusions

In this study, we identified 11 viruses in *A. carmichaelii* plants using HTS and RT-PCR, including AcoPV-1, AcoBPV-1, AcoAV-1, AcVA, CMV, CuLV, ASGV, ChiVMV, TSWV, TVDV, and PLRV. This is the first report of infections with CuLV, ASGV, ChiVMV, PLRV, TVDV, and TSWV in *A. carmichaeli*. In addition, two previously undescribed viruses infecting *A. carmichaeli* were introduced: AcoPV-1 and AcoBPV-1, for which we propose two new species with the formal taxonomic names *Potyvirus aconiti* and *Betapartitivirus aconiti*. 

## Figures and Tables

**Table 1:** Details of viruses identified in individual *A. carmichaelii* samples by RT-PCR.

Virus	Genus	Length (nt)	Isolate Name	Region	GenBank Accession
Aconitum potyvirus 1 (AcoPV-1)	*Potyvirus*	9453	YZYi	Complete genome	MZ389235
Aconitum betapartitivirus 1 (AcoBPV-1)	*Betapartitivirus*	2363	YWDi	Near-complete RNA1	OR096294
		2279	YWDi	Complete RNA2	OR096295
Currant latent virus (CuLV)	*Cheravirus*	6683	YWDi	Complete RNA1	OR096292
		3070	YWDi	Near-complete RNA2	OR096293
Aconite virus A (AcVA)	*Carlavirus*	8833	YWDi	Complete genome	OR096296
Cucumber mosaic virus (CMV)	*Cucumovirus*	657	YWDi	Complete coat protein	MZ389240
Aconitum amalgavirus 1 (AcoAV-1)	*Amalgavirus*	1061	YWDi	Partial fusion protein	OR096297
Apple stem grooving virus (ASGV)	*Capillovirus*	893	YWDi	Partial polyprotein	OR096298
Tobacco vein distorting virus (TVDV)	*Polerovirus*	501	YZYi	Partial coat protein	OR096301
Tomato spotted wilt orthotospovirus (TSWV)	*Orthotospovirus*	504	YZYi	Partial segment M	OR096302
Chilli veinal mottle virus (ChiVMV)	*Potyvirus*	919	YWDi	Partial polyprotein	OR096299
Potato leafroll virus (PLRV)	*Polerovirus*	599	YZYi	Partial capsid protein	OR096300

**Table 2:** Number of positive samples and incidence of individual viruses infecting *A. carmichaelii*.

Virus	County and Collection Year	Zhanyi 2017	Wuding 2020	Wuding 2021	Total
	Sample Number	17	71	54	142
Cucumber mosaic virus (CMV)	Positive samples	11	69	39	119
	Incidence	64.7%	97.2%	72.2%	83.8%
Aconite virus A (AcVA)	Positive samples	0	65	35	100
	Incidence	0	91.6%	64.8%	70.4%
Aconitum potyvirus 1 (AcoPV-1)	Positive samples	15	46	27	88
	Incidence	88.3%	64.8%	50%	62%
Aconitum amalgavirus 1 (AcoAV-1)	Positive samples	2	25	19	46
	Incidence	11.78%	35.2%	35.2%	32.4%
Currant latent virus (CuLV)	Positive samples	0	28	9	37
	Incidence	0	39.5%	16.7%	26.1%
Aconitum betapartitivirus 1 (AcoBPV-1)	Positive samples	0	26	11	37
	Incidence	0	36.6%	20.4%	26.1%
Apple stem grooving virus (ASGV)	Positive samples	0	22	4	26
	Incidence	0	31%	7.4%	18.3%
Tobacco vein distorting virus (TVDV)	Positive samples	7	0	0	7
	Incidence	41.2%	0	0	4.9%
Tomato spotted wilt orthotospovirus (TSWV)	Positive samples	5	0	0	5
	Incidence	29.4%	0	0	3.5%
Chilli veinal mottle virus (ChiVMV)	Positive samples	0	3	0	3
	Incidence	0	4.2%	0	2.1%
Potato leafroll virus (PLRV)	Positive samples	2	0	0	2
	Incidence	11.8%	0	0	1.4%

## Data Availability

The datasets presented in this study can be found in online repositories. The names of the repository/repositories and accession number(s) can be found below: https://www.ncbi.nlm.nih.gov/, MZ389235; OR096292; OR096293; OR096294; OR096295; OR096296; OR096297; OR096298; OR096299; OR096300; OR096301; OR096302; MZ389240.

## Supplementary Materials

The following supporting information can be downloaded at: https://www.mdpi.com/article/10.3390/ijms242115558/s1.

## References

[1] Ma Y., Yang Y.X., Shu X.Y., Huang J., Hou D.B. (2016). *Aconitum carmichaelii* Debeaux, cultivated as a medicinal plant in western China. Genet. Resour. Crop Evol..

[2] Fu Y.P., Zou Y.F., Lei F.Y., Wangensteen H., Inngjerdingen K.T. (2022). *Aconitum carmichaelii* Debeaux: A systematic review on traditional use, and the chemical structures and pharmacological properties of polysaccharides and phenolic compounds in the roots. J. Ethnopharmacol..

[3] Zhou G., Tang L., Zhou X., Wang T., Kou Z., Wang Z. (2015). A review on phytochemistry and pharmacological activities of the processed lateral root of *Aconitum carmichaelii* Debeaux. J. Ethnopharmacol..

[4] Zhao D., Wang J., Cui Y., Wu X. (2012). Pharmacological effects of Chinese herb aconite (fuzi) on cardiovascular system. J. Tradit. Chin. Med..

[5] Liu J., Liang M., Lin T., Zhao Q., Wang H., Yang S., Guo Q., Wang X., Guo H., Cui L. (2023). A LAMP-based toolbox developed for detecting the major pathogens affecting the production and quality of the Chinese medicinal crop *Aconitum carmichaelii*. Plant Dis..

[6] Ma Y., Cao L.L., Yang Y.X., Guan L.L., Gou L.L., Shu X.Y., Huang J., Liu D., Zhang H., Hou D.B. (2017). Genetic diversity and marker–trait association analysis for agronomic traits in *Aconitum carmichaelii* Debeaux. Biotechnol. Biotechnol. Equip..

[7] Cohen J., Zeidan M., Rosner A., Gera A. (2000). Biological and molecular characterization of a new carlavirus isolated from an *Aconitum* sp.. Phytopathology.

[8] Fumiyoshi F., Shin-ichi F., Kouichi S., Masahide I. (2008). Cucumber mosaic virus isolated from *Aconitum* spp. in Japan. J. Gen. Plant Pathol..

[9] Wang R., Chen B., Li Y., Cao M., Ding W. (2021). Complete nucleotide sequence of a new carlavirus infecting *Aconitum carmichaelii* in China. Arch. Virol..

[10] Yang J., Lan P.X., Li J.M., Chen X.J., Tan G.L., Wei T.Y., Li R.H., Li F. (2022). Complete genome sequence of Aconitum amalgavirus 1, a distinct member of the genus *Amalgavirus*. Arch. Virol..

[11] Inoue-Nagata A.K., Jordan R., Kreuze J., Li F., López-Moya J.J., Mäkinen K., Ohshima K., Wylie S.J., Consortium I.R. (2022). ICTV virus taxonomy profile: *Potyviridae* 2022. J. Gen. Virol..

[12] Worrall E.A., Hayward A.C., Fletcher S.J., Mitter N. (2019). Molecular characterization and analysis of conserved potyviral motifs in bean common mosaic virus (BCMV) for RNAi-mediated protection. Arch. Virol..

[13] Vainio E.J., Chiba S., Ghabrial S.A., Maiss E., Roossinck M., Sabanadzovic S., Suzuki N., Xie J., Nibert M., Consortium I.R. (2018). ICTV virus taxonomy profile: *Partitiviridae*. J. Gen. Virol..

[14] Nibert M.L., Ghabrial S.A., Maiss E., Lesker T., Vainio E.J., Jiang D., Suzuki N. (2014). Taxonomic reorganization of family *Partitiviridae* and other recent progress in partitivirus research. Virus Res..

[15] Petrzik K., Koloniuk I., Přibylová J., Špak J. (2016). Complete genome sequence of currant latent virus (genus *Cheravirus*, family *Secoviridae*). Arch. Virol..

[16] Li C., Yoshikawa N., Takahashi T., Ito T., Yoshida K., Koganezawa H. (2000). Nucleotide sequence and genome organization of apple latent spherical virus: A new virus classified into the family *Comoviridae*. J. Gen. Virol..

[17] James D., Upton C. (2005). Genome segment RNA-1 of a flat apple isolate of cherry rasp leaf virus: Nucleotide sequence analysis and RT-PCR detection. Arch. Virol..

[18] Adams I., Glover R., Souza-Richards R., Bennett S., Hany U., Boonham N. (2013). Complete genome sequence of arracacha virus B: A novel cheravirus. Arch. Virol..

[19] Petrzik K., Přibylová J., Špak J., Havelka J. (2015). Partial genome sequence of currant latent virus, a new chera-like virus related to apple latent spherical virus. J. Gen. Plant Pathol..

[20] Khalili M., Candresse T., Brans Y., Faure C., Audergon J.M., Decroocq V., Roch G., Marais A. (2022). The molecular characterization of a new prunus-infecting cheravirus and complete genome sequence of stocky prune virus. Viruses.

[21] Gorbalenya A.E., Koonin E.V., Wolf Y.I. (1990). A new superfamily of putative NTP-binding domains encoded by genomes of small DNA and RNA viruses. FEBS Lett..

[22] Koonin E.V., Dolja V.V., Morris T.J. (1993). Evolution and taxonomy of positive-strand RNA viruses: Implications of comparative analysis of amino acid sequences. Crit. Rev. Biochem. Mol. Biol..

[23] Thompson J.R., Dasgupta I., Fuchs M., Iwanami T., Karasev A.V., Petrzik K., Sanfaçon H., Tzanetakis I., van der Vlugt R., Wetzel T. (2017). ICTV virus taxonomy profile: *Secoviridae*. J. Gen. Virol..

[24] Silva J.M.F., Melo F.L., Elena S.F., Candresse T., Sabanadzovic S., Tzanetakis I.E., Blouin A.G., Villamor D.E.V., Mollov D., Constable F. (2022). Virus classification based on in-depth sequence analyses and development of demarcation criteria using the *Betaflexiviridae* as a case study. J. Gen. Virol..

[25] Beck-Okins A.L., Del Río Mendoza L.E., Burrows M., Simons K.J., Pasche J.S. (2022). Pea seed-borne mosaic virus (PSbMV) risk analysis of field pea based on susceptibility, yield loss, and seed transmission. Plant Dis..

[26] Hansen A.J., Nyland G., McElroy F., Stace-Smith R. (1974). Cherry rasp leaf disease in North America. Phytopathology.

[27] Jones R. (1982). Tests for transmission of four potato viruses through potato true seed. Ann. Appl. Biol..

[28] Brito M., Fernández-Rodríguez T., Garrido M.J., Mejías A., Romano M., Marys E. (2012). First report of *cowpea mild mottle carlavirus* on yardlong bean (*Vigna unguiculata* subsp. sesquipedalis) in Venezuela. Viruses.

[29] Sabanadzovic S., Valverde R.A., Brown J.K., Martin R.R., Tzanetakis I.E. (2009). Southern tomato virus: The link between the families *Totiviridae* and *Partitiviridae*. Virus Res..

[30] Samarskaya V.O., Ryabov E.V., Gryzunov N., Spechenkova N., Kuznetsova M., Ilina I., Suprunova T., Taliansky M.E., Ivanov P.A., Kalinina N.O. (2023). The temporal and geographical dynamics of potato virus Y diversity in Russia. Int. J. Mol. Sci..

[31] Gildow F., Shah D., Sackett W., Butzler T., Nault B., Fleischer S. (2008). Transmission efficiency of cucumber mosaic virus by aphids associated with virus epidemics in snap bean. Phytopathology.

[32] Shah H., Yasmin T., Fahim M., Hameed S., Haque M. (2008). Transmission and host range studies of Pakistani isolate of chilli veinal mottle virus. Pak. J. Bot..

[33] Chen X.J., Luo H.M., Zhang J.Y., Ma Y., Li K.H., Xiong F., Yang Y.H., Yang J.Z., Lan P.X., Wei T.Y. (2022). Synergism among the four tobacco bushy top disease causal agents in symptom induction and aphid transmission. Front. Microbiol..

[34] Olmedo-Velarde A., Wilson J.R., Stallone M., DeBlasio S.L., Chappie J.S., Heck M. (2023). Potato leafroll virus molecular interactions with plants and aphids: Gaining a new tactical advantage on an old foe. Physiol. Mol. Plant Pathol..

[35] Sin S.H., McNulty B.C., Kennedy G.G., Moyer J.W. (2005). Viral genetic determinants for thrips transmission of tomato spotted wilt virus. Proc. Natl. Acad. Sci. USA.

[36] Gadhave K.R., Gautam S., Rasmussen D.A., Srinivasan R. (2020). Aphid transmission of *Potyvirus*: The largest plant-infecting RNA virus genus. Viruses.

[37] Machado Caballero J.E., Lockhart B.E., Mason S.L., Daughtrey M. (2009). Identification and properties of a carlavirus causing chlorotic mottle of florists’ Hydrangea (*H. macrophylla*) in the United States. Plant Dis..

[38] Scholthof K.B.G., Adkins S., Czosnek H., Palukaitis P., Jacquot E., Hohn T., Hohn B., Saunders K., Candresse T., Ahlquist P. (2011). Top 10 plant viruses in molecular plant pathology. Mol. Plant Pathol..

[39] Han J.Y., Park C.H., Seo E.Y., Kim J.K., Hammond J., Lim H.S. (2016). Occurrence of apple stem grooving virus in commercial apple seedlings and analysis of its coat protein sequence. Korean J. Agric. Sci..

[40] Ito T., Namba N., Ito T. (2003). Distribution of citrus viroids and apple stem grooving virus on citrus trees in Japan using multiplex reverse transcription polymerase chain reaction. J. Gen. Plant Pathol..

[41] Lan P.X., He P., Yang J., Zhou G.H., Chen X.J., Wei T.Y., Li C.R., Gu R., Li R.H., Li F. (2022). High-throughput sequencing reveals the presence of novel and known viruses in diseased *Paris yunnanensis*. Front. Microbiol..

[42] Tan S.T., Liu F., Lv J., Liu Q.L., Luo H.M., Xu Y., Ma Y., Chen X.J., Lan P.X., Chen H.R. (2021). Identification of two novel poleroviruses and the occurrence of tobacco bushy top disease causal agents in natural plants. Sci. Rep..

[43] Postler T.S., Rubino L., Adriaenssens E.M., Dutilh B.E., Harrach B., Junglen S., Kropinski A.M., Krupovic M., Wada J., Crane A. (2022). Guidance for creating individual and batch latinized binomial virus species names. J. Gen. Virol..

[44] Li R.H., Mock R., Huang Q., Abad J., Hartung J., Kinard G. (2008). A reliable and inexpensive method of nucleic acid extraction for the PCR-based detection of diverse plant pathogens. J. Virol. Methods.

[45] Waterhouse A.M., Procter J.B., Martin D.M., Clamp M., Barton G.J. (2009). Jalview Version 2-a multiple sequence alignment editor and analysis workbench. Bioinformatics.

[46] Muhire B.M., Varsani A., Martin D.P. (2014). SDT: A virus classification tool based on pairwise sequence alignment and identity calculation. PLoS ONE.

[47] Martin D.P., Murrell B., Golden M., Khoosal A., Muhire B. (2015). RDP4: Detection and analysis of recombination patterns in virus genomes. Virus Evol..

