# Supplementary material for: Virome Analysis of Aconitum carmichaelii Reveals Infection by Eleven Viruses, including Two Potentially New Species
Source: Int J Mol Sci. 2023 Oct 25;24(21):15558. doi: 10.3390/ijms242115558 (PMC10650655; doi:10.3390/ijms242115558)
Supplement: Supplementary file 1 [file ijms-24-15558-s001.zip › ijms-2637330 supplement tables.pdf]

**Table S1** Viral sequences identified by high-throughput sequencing analysis

| Library name | Contig number | Closest related virus | Consensus length (nt) | Total read count | Average coverage | Best match (GenBank accession ) | aa identity (%) | Cover region | Genus |
|---|---|---|---|---|---|---|---|---|---|
| WD-FZ | contig_539 | White clover cryptic virus 2 (WCCV2)，RNA1 | 2,434 | 29,310 | 1, 809 | White clover cryptic virus 2 RNA1 (NC_021094) | 77 | complete *RdRp* gene | *Betapartitivirus* |
| | contig_24 | Cannabis cryptic virus (CanCV), RNA2 | 2,244 | 11,393 | 755 | Cannabis cryptic virus, RNA2 (NC_031130) | 72 | complete *cp* gene | |
| | contig_341 | Currant latent virus (CuLV) | 6,712 | 247,068 | 5, 465 | Currant latent virus, RNA1 (NC_029038) | 75 | complete RNA1 polyprotein gene | *Cheravirus* |
| | contig_62 | | 2,270 | 114,114 | 7, 402 | Currant latent virus, RNA2 (KC935375) | 75 | partial RNA2 polyprotein gene | |
| | contig_30 | Aconite virus A (AcVA) | 5,428 | 1,166,397 | 31, 210 | Aconite virus A (MN944106) | 72 | nearly complete *RdRp* gene | *Carlavirus* |
| | contig_15, 527 | Cucumber mosaic virus (CMV) | 3,371 | 1,810,218 | 75, 124 | Cucumber mosaic virus RNA1 (LC593244) | 98 | almost complete RNA1 | *Cucumovirus* |
| | contig_61 | | 3,053 | 699,777 | 30, 895 | Cucumber mosaic virus RNA2 (LC593245) | 99 | almost complete RNA2 | |
| | contig_82 | | 2,217 | 3,575,260 | 184, 183 | Cucumber mosaic virus RNA3 (LC593246) | 98 | almost complete RNA3 | |
| | contig_1317 | Apple stem grooving virus (ASGV) | 6,509 | 24,272 | 540 | Apple stem grooving virus (KU947036) | 97 | almost complete genome | *Capillovirus* |
| ZY-FZ | contig_6, 287 | Tulip breaking virus (TBV) | 9,479 | 2,188,397 | 135, 266 | Tulip breaking virus (MH886517) | 56 | almost complete genome | *Potyvirus* |
| | contig_1, 934 | Cucumber mosaic virus (CMV) | 2,856 | 1,975,751 | 89,806 | Cucumber mosaic virus RNA1 (KC527787) | 99 | almost complete RNA1 | *Cucumovirus* |
| | contig_362 | | 2,963 | 256,569 | 4,082 | Cucumber mosaic virus RNA2 (KC52769) | 100 | almost complete RNA2 | |
| | contig_352 | | 2,207 | 2,568,895 | 107,037 | Cucumber mosaic virus RNA3 (EF216865) | 98 | almost complete RNA3 | |
| | contig_31 | Chilli veinal mottle virus | 7,930 | 545,473 | 6, 852 | chilli veinal mottle virus isolate | 98 | nearly complete | *Potyvirus* |

| | | | | | | | | |
|---|---|---|---|---|---|---|---|---|
| | isolate (ChiVMV) | | | | (MT974520) | | genome | |
| contig_5, 396 | | 8,894 | 2,895,365 | 65,803 | tomato spotted wilt orthotospovirus segment L (MF805766) | 99 | almost complete genome | |
| contig_8, 094 | Tomato spotted wilt orthotospovirus (TSWV) | 2,985 | 947,758 | 37,910 | tomato spotted wilt orthotospovirus segment S (HQ402595) | 99 | almost complete genome | *Orthotospovirus* |
| contig_ 617 | | 4,840 | 589,258 | 13,392 | tomato spotted wilt orthotospovirus segment M (JF960236) | 99 | almost complete genome | |
| contig_986 | Tobacco vein distorting virus (TVDV) | 2,418 | 8,637 | 148 | tobacco vein distorting virus (EF529624) | 88 | complete *RTD* gene | *Polerovirus* |
| contig_943 | Potato leafroll virus （PLRV） | 2157 | 5,768 | 94 | potato leafroll virus (NC_001747) | 88 | partial *RTD* gene | *Polerovirus* |

**Table S2** Identity of the RNA1 and RNA2 encoded polyproteins between CuLV-YWDi and other cheraviruses

| | | | | | |
|---|---|---|---|---|---|
| CuLV-YWDi RNA1 | CULV (KT692952) | ALSV (AB030940) | CRLV (MK952187) | AVB (MK153131) | StPV (OP328251) |
| | 75.7% | 66.4% | 63% | 33.8% | 27.6% |
| CuLV-YWDi RNA2 | CuLV (KT692953) | ALSV (AB030941) | (MK952188) CRLY | AVB (MK153132) | StPV (OP328252) |
| | 74.7% | 68.7% | 65.5% | 22.9% | 22.6% |

**Table S3** Primers used for detection the viruses infecting *Aconitum carmichaelii*

| Virus | Primer name* | Primer sequence (5'-3') | Size (nt) | Tm（℃） |
|---|---|---|---|---|
| AcoPV-1 | FZPoF8659 | TTGAGGCAGATTAGCTCCAAGC | 532 | 57 |
| | FZPoR9191 | TATTGCGTAAGGCTGCTGCCTTC | | |
| AcoBPV-1 | WCCV2R2F1464 | ACACAGCTCCAAGCGTTCTTGTCC | 494 | 58 |
| | WCCV2R1R1931 | TTCGGACATGCCAGGAACAGC | | |
| CuLV-YWDi | CuLVR1F61 | TTCGTTTCCCAACTCTGCTACC | 665 | 55 |
| | CuLVR1R725 | AACAACGCCACGTGGCATATCG | | |
| AcoAV-1 | FZAVF1900 | ATGATTCCCAGTGGCTCTCTC | 1100 | 57 |
| | FZAVR3000 | TGTAGTCCACGCTGCATATGC | | |
| AcVA | AcVAF109 | ATATCGTAGCCCACTTGAGG | 905 | 55 |
| | AcVAR994 | TGCAAGTTGTGGCTTCGAACC | | |
| | AcVAF201 | ACTATGCACTTGAGGCTCATG | 714bp | 55 |
| | AcVAR914 | TAACGTGGTGAGCGAATTTGC | | |
| CMV | CMVCPuF | TCTCATGGATGCTTCTCCGCG | 880 | 55 |
| | CMVCPuR | CCGTAAGCTGGATGGACAACC | | |
| ASGV | ASGVF5431 | TCTGGAAGAAGGTGCACCAAACG | 918 | 56 |
| | ASGVR6326 | TGCAAGACCGCGACCAAATTTG | | |
| ChiVMV | ChiVMVdF | GGATAGAGCTGARCARCCAG | 920 | 57 |
| | ChiVMVdR | CTTTGAAGCCCATATCTTGGC | | |
| TSWV | TSWVMF1829 | TGATAAGTAGATAGAGAGCAAGC | 503 | 55 |
| | TSWVMR2332 | TGAACTGATAGCTCATGGACC | | |
| TVDV | TVDVdF | ATGGTGACTCTGAAGGATCCTGC | 534 | 57 |
| | TVDVdR | TGGTCAACATACGCTTCGTCAGTG | | |
| PLRV | PLRV-CP-F | ATGAGTACGGTCGTGGTTAAAG | 600 | 57 |
| | PLRV-CP-R | ATGGTGACTCTGAAGGATCCTGC | | |
| AcLV | AcLVdF | AGTTCAACGAAGGTCTCAAAGG | 971 | 54 |
| | AcLVdR | TCTCTGGTTAATTCAGGTCCATTGC | | |

* F: Forward primer; R: Reverse primer; primers of AcVAF201 and AcVAR914 were used to detection AcVA in *A. carmichaelii* seedlings.

**Table S4** Single or mixed viral-infection rates in *Aconitum carmichaelii* detected by RT-PCR

| Viral viruses | Numbers of virus-positive sample | Detection rate (%) |
|---|---|---|
| 1 virus | 9 | 6.3 |
| 2 viruses | 19 | 13.4 |
| 3 viruses | 34 | 23.9 |
| 4 viruses | 40 | 28.2 |
| 5 viruses | 22 | 15.5 |
| 6 viruses | 6 | 4.2 |
| 7 viruses | 2 | 1.4 |
| No virus | 10 | 7.0 |
| Total | 142 | |

Note: A total of 132 in 142 samples were virus-positive

**Table S5** Viruses involved in single or mixed infection in *Aconitum carmichaelii*

| order | viruses | Single or mixed viral-infection types | Positive numbers | Detection rate (%) |
|---|---|---|---|---|
| 1 | 1 virus | AcoPV-1 | 4 | 2.8 |
| 2 | 1 virus | CuLV | 2 | 1.4 |
| 3 | 1 virus | CMV | 1 | 0.7 |
| 4 | 1 virus | TSWV | 1 | 0.7 |
| 5 | 1 virus | TVDV | 1 | 0.7 |
| 6 | 2 viruses | AcoPV-1+CMV | 8 | 5.6 |
| 7 | 2 viruses | ACVA+CMV | 6 | 4.2 |
| 8 | 2 viruses | AcoAV-1+CMV | 2 | 1.4 |
| 9 | 2 viruses | CuLV +CMV | 1 | 0.7 |
| 10 | 2 viruses | ACVA+ASGV | 1 | 0.7 |
| 11 | 2 viruses | AcoPV-1+TVDV | 1 | 0.7 |
| 12 | 3 viruses | AcoPV-1+ACVA+CMV | 12 | 8.5 |
| 13 | 3 viruses | AcoAV-1+ACVA+CMV | 7 | 4.9 |
| 14 | 3 viruses | AcoBPV-1+ACVA+CMV | 4 | 2.8 |
| 15 | 3 viruses | AcoPV-1+CMV+TVDV | 2 | 1.4 |
| 16 | 3 viruses | AcoPV-1+AcoAV-1+CMV | 2 | 1.4 |
| 17 | 3 viruses | CuLV +ACVA+CMV | 2 | 1.4 |
| 18 | 3 viruses | AcoPV-1+TSWV+TVDV | 1 | 0.7 |
| 19 | 3 viruses | AcoPV-1+CMV+TSWV | 1 | 0.7 |
| 20 | 3 viruses | AcoAV-1+CMV+ChiVMV | 1 | 0.7 |
| 21 | 3 viruses | AcoBPV-1+CuLV +AcoAV-1 | 1 | 0.7 |
| 22 | 3 viruses | AcoPV-1+AcoAV-1+ACVA | 1 | 0.7 |
| 23 | 4 viruses | AcoPV-1+AcoBPV-1+ACVA+CMV | 8 | 5.6 |
| 24 | 4 viruses | AcoPV-1+AcoAV-1+ACVA+CMV | 8 | 5.6 |
| 25 | 4 viruses | AcoPV-1+CuLV +ACVA+CMV | 7 | 4.9 |
| 26 | 4 viruses | AcoPV-1+ACVA+CMV+ASGV | 4 | 2.8 |
| 27 | 4 viruses | AcoBPV-1+AcoAV-1+ACVA+CMV | 3 | 2.1 |

| | | | | |
|---|---|---|---|---|
| 28 | 4 viruses | AcoBPV-1+ACVA+CMV+ASGV | 2 | 1.4 |
| 29 | 4 viruses | CuLV +ACVA+CMV+ASGV | 2 | 1.4 |
| 30 | 4 viruses | AcoPV-1+CMV+TVDV+PLRV | 1 | 0.7 |
| 31 | 4 viruses | AcoPV-1+AcoBPV-1+TSWV+TVDV | 1 | 0.7 |
| 32 | 4 viruses | CuLV +AcoAV-1+ACVA+CMV | 1 | 0.7 |
| 33 | 4 viruses | AcoPV-1+AcoAV-1+ACVA+ASGV | 1 | 0.7 |
| 34 | 4 viruses | AcoBPV-1+CuLV +ACVA+CMV | 2 | 1.4 |
| 35 | 5 viruses | AcoPV-1+AcoBPV-1+AcoAV-1+ACVA+CMV | 5 | 3.5 |
| 36 | 5 viruses | AcoPV-1+CuLV +AcoAV-1+ACVA+CMV | 3 | 2.1 |
| 37 | 5 viruses | AcoPV-1+CuLV +ACVA+CMV+ASGV | 3 | 2.1 |
| 38 | 5 viruses | AcoPV-1+AcoAV-1+ACVA+CMV+ASGV | 3 | 2.1 |
| 39 | 5 viruses | AcoPV-1+AcoBPV-1+CuLV +ACVA+CMV | 2 | 1.4 |
| 40 | 5 viruses | AcoBPV-1+CuLV +ACVA+CMV+ASGV | 2 | 1.4 |
| 41 | 5 viruses | AcoPV-1+AcoAV-1+CMV+TSWV+PLRV | 1 | 0.7 |
| 42 | 5 viruses | AcoPV-1+AcoBPV-1+ACVA+CMV+ASGV | 1 | 0.7 |
| 43 | 5 viruses | AcoPV-1+AcoAV-1+ACVA+CMV+ChiVMV | 1 | 0.7 |
| 44 | 5 viruses | CuLV +AcoAV-1+ACVA+CMV+ChiVMV | 1 | 0.7 |
| 45 | 6 viruses | AcoPV-1+AcoBPV-1+CuLV + ACVA+CMV+ASGV | 3 | 2.1 |
| 46 | 6 viruses | AcoPV-1+AcoBPV-1+CuLV +AcoAV-1+ACVA+ CMV | 1 | 0.7 |
| 47 | 6 viruses | AcoPV-1+CuLV +AcoAV-1+ACVA+CMV+ASGV | 1 | 0.7 |
| 48 | 6 viruses | AcoBPV-1+CuLV +AcoAV-1+ACVA+ CMV+ASGV | 1 | 0.7 |
| 49 | 7 viruses | AcoPV-1+AcoBPV-1+ CuLV +AcoAV-1+AcVA+CMV+ASGV | 2 | 1.4 |
| | No virus | | 10 | 7.0 |
| total | | | 142 | |

**Table S6** Primers used for amplification of the complete nucleotide sequence of AcoPV-1

| Primer name* | Primer sequence (5’-3’) | Size (nt) | Tm (°C) | Note |
|---|---|---|---|---|
| PotyvirusF98 | AAGCACACATAAGCACAATGGCG | 1603 | 58 | |
| PotyvirusR1701 | ACATTCGGATTTCTCCTGATTACG | | | |
| PotyvirusF1588 | TTCAAATGGCGCCAGCGCGC | 1579 | 58 | |
| PotyvirusR3167 | AGCTGCAACAAGACACACATCAC | | | |
| PotyvirusF3099 | AAGAAGCGCAATGGCAGATCTC | 1553 | 58 | |
| PotyvirusR4555 | TTCAGGCCAAAATCAACAACCGC | | | |
| PotyvirusF4442 | GATACCAACAAGTGGAACTCAC | 1544 | 58 | |
| PotyvirusR5986 | TTACTGAGTCGATGTCCAGCTC | | | |
| PotyvirusF5845 | ACTTTTGCACGGTATGTCGATCC | 1614 | 58 | |
| PotyvirusR7459 | TCATTGAGCCATTCCACACTCC | | | |
| PotyvirusF7305 | AGCATTGAATATGAAGGCAGCAGTC | 1467 | 58 | |
| PotyvirusR8772 | ATCCTACTGTGCATGTGTTGCTC | | | |
| PotyvirusF8659 | TTGAGGCAGATTAGCTCCAAGC | 532 | 58 | |
| PotyvirusR9191 | TATTGCGTAAGGCTGCTGCCTTC | | | |
| PotyvirusF8971 | AATGCACAACCCACACTAAGGC | ~540 | 58 | For 3’-end |

| | | | | |
|---|---|---|---|---|
| Vial9（PCR） | GACCACGCGTATCGATGTCGAC | | | |
| Vial8（RT） | GACCACGCGTATCGATGTCGACTTTTTTTTTTTTTTT V （V = A, C or G） | | | |
| PotyvirusR224 | ATGTTGGATTCGTAGTTGGCAATAGC | ~220 | 65 | For 5’ RACE |
| PotyvirusR386 | TTCTTGAGAGCCGCATCCTGGGCCTC | ~380 | 60 | |

* F: Forward primer; R: Reverse primer

**Table S7** Primers used for amplification of the complete nucleotide sequence of CuLV-YWDi

| Primer name* | Primer sequence (5’-3’) | Size (nt) | Tm (℃) | Segment | Note |
|---|---|---|---|---|---|
| CheravirusR1R191 | ACATGTGTCCTCTGGAGAGGAG | ~190 | 58 | RNA1 | For 5’ RACE |
| CheravirusR1R232 | ATACGCATTGACCTCTGGAGAGAGGC | ~220 | 62 | | |
| CheravirusR1F61 | TTCGTTTCCCAACTCTGCTACC | 660 | 58 | | |
| CheravirusR1R725 | AACAACGCCACGTGGCATATCG | | | | |
| CheravirusR1F550 | ATCAACGCAGACAGGCTCTGC | 1471 | 58 | | |
| CheravirusR1R2033 | AAGGCATCCATCCAGCTAACC | | | | |
| CheravirusR1F1901 | AGGTGCAAAAGAAACGGTTGTGC | 1592 | 58 | | |
| CheravirusR1R3493 | AACATATACAGGGCAAACTTGATC | | | | |
| CheravirusR1F3320 | TGCATCATTCCCTCAATTCGAGGC | 1729 | 58 | | |
| CheravirusR1R5031 | ATGTGCCAATCTCCAGGAGATCC | | | | |
| CheravirusR1F4798 | ATGCTGAGTCGCTAGTTATGCAG | 1537 | 58 | | |
| CheravirusR1R6334 | TGTGAGACCACCCAATCTGTAG | | | | |
| Vial9（PCR） | GACCACGCGTATCGATGTCGAC | ~400 | 56 | | For 3’-end |
| Vial8（RT） | GACCACGCGTATCGATGTCGACTTTT TTTTTTTTTTTV （V = A, C or G） | | | | |
| CheravirusR1F6179 | TGGTCTCTATGCAGTTTAAAAGCTCG | | | | |
| CheravirusR2R178 | ACAGGATTCAACCAACCGCTGGTGGC | ~200 | | RNA2 | For 5’ RACE |
| CheravirusR2F150 | ACTTCCCTCCCTTCTTCTAAC | 330 | 54 | | |
| CheravirusR2R480 | TCATCTTTGGCCAAGTAGGTGG | | | | |
| CheravirusR1F362 | AATGCTGGCACGTCTCCGTGC | 1652 | 58 | | |
| CheravirusR1R2012 | AGACGGTGTCCCATTCCTACTAGC | | | | |
| CheravirusR2F1846 | TCTACACCTTCCCTTCAATAGTTG | 1274 | 58 | | |
| CheravirusR2R3120 | ATGATTAGCACGGACCCAAAGTTG | | | | |
| CheravirusR2F2767 | ATGTGGTTGCGCACCTATCGAGTTGC | ~400 | 60 | | For 3’-end |
| Vial9（PCR） | GACCACGCGTATCGATGTCGAC | | | | |
| Vial8（RT） | GACCACGCGTATCGATGTCGACTTTT TTTTTTTTTTTV （V = A, C or G） | | | | |

* F: Forward primer; R: Reverse primer

**Table S8** Primers used for amplification of the complete nucleotide sequence of AcoBPV-1

| Primer name* | Primer sequence (5’-3’) | Size (nt) | Tm (°C) | Segment | Note |
|---|---|---|---|---|---|
| BetapartitivirusR1R200 | AGTAGAATCAAGAACAGCAGTGGGATCAC | ~200 | 62 | RNA1 | For 5’ RACE |
| BetapartitivirusR1F23 | ACATTCGTCTCCAGTATCAAGC | 2072 | 58 | | |
| BetapartitivirusR1R2094 | TTGCTAGATCAGGCATTCCATC | | | | |
| Vial9（PCR） | GACCACGCGTATCGATGTCGAC | ~400 | 60 | | For 3’-end |
| Vial8（RT） | GACCACGCGTATCGATGTCGACTTTTTTTTTTTTTTTV （V = A, C or G） | | | | |
| BetapartitivirusR1F2007 | TCTCACTATAAACCAGACGTTCGC | | | | |
| BetapartitivirusR2R233 | TGAAGAATCAGTACGAGCAGCAGTATTGG | ~250 | 62 | RNA2 | For 5’ RACE |
| BetapartitivirusR2F80 | TCTAACGATGTCTACCTCATCTAC | 1852 | 58 | | |
| BetapartitivirusR1R1931 | TTCGGACATGCCAGGAACAGC | | | | |
| BetapartitivirusR2F1815 | ATGACTCCAATCACTCCTGTGAATG | ~400 | 56 | | For 3’-end |
| Vial9（PCR） | GACCACGCGTATCGATGTCGAC | | | | |
| Vial8（RT） | GACCACGCGTATCGATGTCGACTTTTTTTTTTTTTTTV （V = A, C or G） | | | | |

* F: Forward primer; R: Reverse primer

**Table S9** Primers used for amplification of the complete nucleotide sequence of AcVA-YWDi

| Primer name* | Primer sequence (5’-3’) | Size (nt) | Tm (°C) | Note |
|---|---|---|---|---|
| AcVAR148 | TACCTATCGACGGCTGTGGCAGAG | ~150 | 62 | For 5’ RACE |
| AcVAF27 | AGATAAGAGCTGAACATACACGAC | 1543 | 58 | |
| AcVAR1569 | AGCAATGTGACTTCACTAGCAGC | | | |
| AcVAF1462 | TACTGCTGGGACAAGCTATGGC | 1504 | 58 | |
| AcVAR2965 | TACATCTATCCTCACAAAGCTCTC | | | |
| AcVAF2851 | ACCTAGTGCTGTGCCTATGGAC | 1524 | 58 | |
| AcVAR4374 | TCAGCACTACAACCAGTGAGCTC | | | |
| AcVAF4257 | ATTATTGGCCGACTGCCGTGC | 1413 | 58 | |
| AcVAR5670 | AGGAAAGTGAACAACATATTGGCC | | | |
| AcVAF5410 | TGTCGCTTTGCACCTTACATGC | 1461 | 58 | |
| AcVAR6871 | AGGTCACAAGAGTTACCACGTC | | | |
| AcVAF6785 | ATTACGTAAGCACTGCGTGCC | 1482 | 58 | |
| AcVAR8267 | ATTCAAACACGTCGAAAGCAGC | | | |
| AcVAF7900 | ACAACTTGCGAGGCGACACCAC | ~950 | 58 | For 3’-end |
| Vial9（PCR） | GACCACGCGTATCGATGTCGAC | | | |
| Vial8（RT） | GACCACGCGTATCGATGTCGACTTTTTTTTTTTTTTTV （V = A, C or G） | | | |

* F: Forward primer; R: Reverse primer
